# The Daily Challenges of Caring for Grandchildren: A Daily Analysis of Emotional and Physical Reactivity to Stressors

**DOI:** 10.1093/geroni/igab046.3444

**Published:** 2021-12-17

**Authors:** Elizabeth Hahn Rickenbach, Janelle Fassi

**Affiliations:** Saint Anselm College, Manchester, New Hampshire, United States

## Abstract

Grandparents are increasingly providing extensive and custodial care for their grandchildren. Many factors have contributed to a societal rise in caregiving among grandparents, including addiction, incarceration, dual-income families, and the cost of childcare. Past work has highlighted positive effects of grandparenting (e.g. reduced dementia risk); however, research is limited that examines the day to day challenges grandparent caregivers experience. The goal of this research was to examine daily experiences of stressors, positive events, physical symptoms, and daily mood of grandparent caregivers. Participants (n=18 grandparent caregivers) filled out a diary survey for five consecutive days that measured daily stressors and positive events. A total of 90 diaries were completed. Stressors were reported on 97.6% of days. Multilevel analysis examining emotional and physical reactivity to daily events showed that, controlling for age and gender, on days when participants reported more stressors than average, they reported higher negative affect (p=.019), lower positive affect (p=.003) and more physical symptoms (p=.002). Positive events were not significantly associated with daily mood or daily physical symptoms. Overall, the findings supported the hypothesis that grandparent caregivers experience emotional and physical reactivity to the daily challenges they experience. Future research should examine resources and supports to reduce the impact of daily stressors, as well as the particular challenges among underrepresented groups, particularly Black and Latino grandparents, who provide disproportionate levels of care for their grandchildren. The current study highlights the potential vulnerability and daily needs for support among grandparents who provide regular and custodial care for their grandchildren.

